# The Development of Co-Occurrent Anxiety and Externalizing Problems from Early Childhood: a Latent Transition Analysis Approach

**DOI:** 10.1007/s10802-021-00865-2

**Published:** 2021-09-09

**Authors:** Aimé Isdahl-Troye, Paula Villar, Beatriz Domínguez-Álvarez, Estrella Romero, Kirby Deater-Deckard

**Affiliations:** 1 Department of Clinical Psychology and Psychobiology, University of Santiago de Compostela, Santiago de Compostela, Spain; 2 Psychological and Brain Sciences, University of Massachusetts Amherst, Amherst, USA

**Keywords:** Anxiety, Externalizing problems, Co-occurrence, Early development, Latent transition analysis

## Abstract

Research into co-occurrent internalizing and externalizing problems during childhood is flourishing. In particular, investigation on the association between anxiety and externalizing problems has yielded mixed findings, focused mainly on the issue of which problem might precede the other, and what role anxiety plays with respect to externalizing problems. Relatively little attention has been paid to the developmental patterns of these behaviors from early childhood, despite the potential of such knowledge to fully delineate etiological models of co-occurrence. This study aimed to examine the longitudinal association of anxiety and externalizing problems in a community sample of preschoolers (*ELISA Project*; N = 2,341; 48.2% girls), by identifying empirically derived profiles and then describing their change and stability through the use of Latent Transition Analysis. Gender differences were explored. Four different profiles were identified: “typically developing”, “mainly anxious”, “modestly externalizing” and “co-occurrent”. Membership in these profile groups showed high stability over a two-year period. However, children in the “co-occurrent” profile group were the most likely to show changes, predominantly towards “modestly externalizing”. Furthermore, a significant gender difference for transitions towards the “co-occurrent” profile group was found, with girls showing less likelihood of being assigned to such profile. These findings show that it is possible to identify an early persistent course of co-occurrent anxiety and externalizing problems, as well as observe changes in co-occurrence towards a simpler externalizing behavioral expression. Further research should explore predictors of group membership and changes in membership, that are malleable and therefore open to preventative intervention.

**Supplementary Information:**

The online version contains supplementary material available at 10.1007/s10802-021-00865-2.

Anxiety and externalizing problems such as oppositional/defiant (OD) and attentional deficit/hyperactive (ADH) disorder symptoms, are among the most prevalent forms of early psychopathology (Egger & Angold, 2006). Problems like these have been seen to develop in stable courses from early childhood (Briggs-Gowan et al., 2006), as well as to co-occur at higher rates than would be expected by chance, in both community and clinic-based samples (Angold et al., 1999; Russo & Beidel, 1994). The research literature has recognized that co-occurring anxiety and externalizing problems are a real phenomenon (Bubier & Drabick, 2009) – which is not merely due to methodological artifacts (Franco et al., 2007); this has important nosological and clinical implications. For example, children who experience this co-occurrent behavior have been found to have a high-risk profile in terms of high symptom severity and significant rates of functional deterioration (Fraire & Ollendick, 2013; Franco et al., 2007; Yoo et al., 2009), even in early childhood (Martín et al., 2014).

Nevertheless, there is a relative paucity of research on the co-occurrence of anxiety and externalizing problems as reported in the major prior literature reviews in this field (Bubier & Drabick, 2009; Russo & Beidel, 1994). One major reason for this is the presence of mixed findings that have been difficult to integrate in a whole framework. More recent findings on the association of anxiety and externalizing problems continue to show mixed results regarding the co-occurrence of anxiety with OD symptoms (Lavigne et al., 2014), ADH symptoms (Becker et al., 2012; Sciberras et al., 2014), or both OD and ADH (Danforth et al., 2019; Murray et al., 2018). Note that these studies frequently included both measures of OD and ADH symptoms, in order to control for the other’s potential effects. In addition, other variables such as subclinical measures of anxiety and externalizing behaviors (i.e., reactive aggression) have been considered (Becker et al., 2012; Murray et al., 2018), in order to arrive at even more precise and narrowly defined details of the association between anxiety and externalizing problems. Despite progress in this area of work, debate remains about the role that anxiety plays with respect to the development of externalizing problems, and about which problem precedes the other, as researchers strive to explain their co-occurrence. In light of this, research is calling for more longitudinal studies which examines the patterns and test the underlying mechanisms involved in the developmental emergence of co-occurring anxiety and externalizing problems over time (Becker et al., 2012; Murray et al., 2018).

Of particular importance, advancing our understanding of the developmental emergence of co-occurring internalizing and externalizing problems is a very active research emphasis in the field (e.g., Deutz et al., 2020; Oh et al., 2020). From this rapidly growing literature, several hypotheses about the mechanisms underlying co-occurrence have been derived—and most of these were tested with regard to the association between anxiety and externalizing problems. Some studies have suggested that co-occurrence might emerge because of common processes underlying internalizing and externalizing problems (Angold et al., 1999; Cosgrove et al., 2011), raising the idea that there is shared risk across both types of problems (Aldao et al., 2016; Shi et al., 2020). On this matter, there is some evidence of common risk factors among overlapping anxiety and externalizing problems, within the child (e.g., low emotional regulation, information processing deficits), and within the child’s socio-environmental context (e.g., parental greater use of psychological control, less positive emotional expressiveness) (Eisenberg et al., 2010; Fraire & Ollendick, 2013).

In contrast, a notably distinct view is presented in the *accumulation hypothesis*, which proposes that there are causal effects of one of these problem types on the emergence of the other problem type—which becomes co-occurring with the initial type of problem. Several studies have shown that externalizing problems during childhood can lead to future internalizing problems (Fanti et al., 2019; Gilliom & Shaw, 2004), with findings identifying anxiety problems as being particularly important (Wichstrøm et al., 2017). There is support for this hypothesis among studies examining the interrelatedness of anxiety and externalizing problems, although some findings have found an enhancing effect of prior anxiety on later externalizing problems. The distinction in the temporal pattern may be due to development. Bubier and Drabick (2009) have stated that externalizing problems during early childhood may be more likely to confer risk for anxiety problems, whereas anxiety in childhood and adolescence may predict emergence of adolescent externalizing problems. Clearly, the expressions of anxiety and components of externalizing problems can change in their association within and across age points over the course of child and adolescent development.

The lion’s share of longitudinal research in this field has examined the prospective associations between anxiety and externalizing problems during childhood and adolescence, yet only a small number of these longitudinal studies have specifically addressed the developmental course of co-occurrent anxiety and externalizing problems. Furthermore, there have been few attempts to examine such patterns of covariation in early childhood (Lavigne et al., 2014; Martín et al., 2014). Thus, the developmental progression of co-occurrent anxiety and externalizing behaviors across early childhood still remains somewhat uncharted territory. One of the challenges for studying co-occurrence at young ages is that symptoms covariation may be simply due to low symptom specificity, which is a common feature of problem behaviors in early childhood (Nottelmann & Jensen, 1995). These early co-occurrent patterns might be progressively redefined into expressions with lower levels of co-occurrence as children develop and interact with their environment (i.e., the *differentiation hypothesis*).

In support of the differentiation hypothesis, some studies exploring problem development across early childhood have shown higher levels of behavioral transitions and discontinuities over time (Finsaas et al., 2018; Jobs et al., 2019) in comparison to later developmental periods (Wichstrøm et al., 2017). However, there is also some evidence of a lasting pattern of problems starting during early childhood for some children (Briggs-Gowan et al., 2006; Finsaas et al., 2018). Wichstrøm et al. (2017) posited the idea that invariant factors (e.g., genes, stable parenting environments) might represent the main predictors of continuities in behavioral problems. Thus, if there were common factors underlying the emergence of co-occurring behavioral and emotional problems, and if they were stable during development, persistent co-occurrent patterns might be observed even from early in development. Accordingly, Willner et al. (2016) found that preschoolers showing a co-occurrent profile of internalizing and externalizing problems had high probabilities to remain in that profile group across early childhood.

In addition to consideration of the developmental period differences in hypothesized patterns of differentiated and co-occurring problems, researchers also must contemplate child gender. One hypothesis has suggested that girls might exhibit less externalizing problems than boys, but girls’ existent severe externalizing behaviors could increase the risk of co-occurring conditions (e.g., anxiety problems) (Euler et al., 2015; Polier et al., 2012). This hypothesis has gained support mainly in studies spanning late childhood and adolescence, but the findings have not always been consistent (Marmorstein, 2007; Munkvold et al., 2011). The same applies for research during early childhood, with even fewer studies that also showed mixed results. Among these early childhood studies, some have shown that co-occurring patterns of externalizing and internalizing problems are gender-invariant in preschoolers (Martín et al., 2014; Wang & Yan, 2019), but others have reported the presence of more boys in subgroups with high externalizing problems (which includes the co-occurring pattern with internalizing problems) by the end of early childhood (Basten et al., 2013, 2016; Shi et al., 2020). Thus, as problem expressions change over development, gender may have a distinct effect on differentiated or co-occurring anxiety and externalizing problems depending on the developmental period (Marmorstein, 2007). Therefore, additional research is needed to clarify any potential gender differences in the early development of co-occurring patterns of externalizing and anxiety problems (Bubier & Drabick, 2009).

There are not many studies that have specifically explored the progression of co-occurrent symptoms over childhood, but this small literature includes a body of research employing latent profile and transition models to describe pattens of stability and change between and among different behavioral classes or profiles (Basten et al., 2016; McElroy et al., 2017; Willner et al., 2016). Such models have the advantage of addressing behavioral change from a person-centered approach, which in comparison with the more common variable-centered approach may provide a more detailed view of how behavioral developmental patterns differ among children. The variable-centered approach assumes children are homogeneous with respect to how such behaviors are related. Therefore, a longitudinal and variable-centered study design would account for universal patterns of behavioral change on a given population of children. A person-centered approach complements variable-centered findings by examining differences among subgroups of children in terms of how distinct behaviors are associated. Note that the behavioral transitions and discontinuities observed during early childhood might be due in part to children not all sharing the same growth pattern of problem behavior across stages of development. The differing patterns in growth can be better described and understood if 1) models identify subgroups who share a specific pattern of association between behaviors, and 2) longitudinally examine children’s probabilities of changing between subgroups over time.

To this end, latent profile and transition models present specific advantages for examining the developmental patterns of concurrent anxiety and externalizing problems over childhood. First, previous studies have already combined measures of anxiety and externalizing to define groups, mostly establishing statistical or clinical cut-off points (e.g., Becker et al., 2012; Danforth et al., 2019; Yoo et al., 2009). However, these investigations rarely use both measures of externalizing problems to define the groups. This is probably due to the intent to focus on the association of anxiety with only one externalizing problem, but also because of the difficulty in identifying a subgroup with intense problems on multiple indicators. Latent profile models account for complex multidimensional aspects of individual functioning, identifying behavioral patterns using multiple theorical-related variables. Through this approach individuals are classified into a set of underlying subgroups by using a statistically based method rather than thresholds or cut-off points. This is an effective tool for identifying subgroups that are internally homogeneous and externally heterogeneous (Petersen et al., 2019), even if these subgroups are small. Thus, latent profile approach may be particularly useful for examining the early development of co-occurrent behaviors, due to the challenge of identifying clinical forms at these young ages. Second, most of the prior relevant studies have employed clinical or high-risk samples instead of large community samples. Community samples have the advantage of being able to include many more participants (i.e., greater statistical power) and greater variability in symptoms that may reflect early signs of the development of later, more severe problems. Latent profile models are well suited for large community samples, because the greater variability in symptoms and large sample make it feasible to identify profile solution that best fit the data. Third, the latent transition modeling approach examines different behavioral patterns of changes in profile membership over development. Thus, this model approach may provide a detailed view of the heterogeneity within the course of these different behavioral patterns. In other words, this addresses the diversity of trajectories of profile membership, with some subgroups of children remaining in the same profile class while others shift to more complex (or simpler) presentations of symptoms in different classes. This is essential for testing the accumulation and differentiation hypotheses regarding symptom co-occurrence.

To our knowledge, there is no previous study that has used latent profile and transition models to examine anxiety and externalizing co-occurrence across early childhood. Thus, our main goal in the current study was to examine the association of anxiety and externalizing problems during early childhood using a longitudinal study design and applying a person-centered analysis method. Our first aim was to identify homogeneous subgroups or profiles based on combinations of anxiety and externalizing problems (OD and ADH) by employing a latent profile analysis (LPA) model. On the basis of previous studies exploring the associations between internalizing and externalizing problems using LPA (e.g., Basten et al., 2016; Willner et al., 2016), we expected to identify at least four profiles based on anxiety and externalizing problems, that would be quantitatively (in terms of degree of symptoms severity) and qualitatively (in terms of the distinct combination of symptoms in each profile group) different. Among these, we anticipated that the profile group showing the highest rates of co-occurrence also would exhibit the highest symptom levels compared to the other profile groups. Our second aim was to analyze stability and change in membership profile status over the course of two annual follow-up assessments following an initial assessment during the preschool years. In accordance with prior evidence of changes in problems during early childhood (compared to later childhood and adolescence, when problem behaviors are much more stable over time), along with the differential hypothesis, we expected that changes in profile membership would tend to shift over time toward lower intensity, more differentiated, less co-occurring symptom profile membership (Basten et al., 2016). However, again based on former studies, we also anticipated that some young children would show a shift over time into higher co-occurrence along with more intense symptoms (Willner et al., 2016), which would be consistent with the accumulation hypothesis. The third aim of the study was to test the predictive effects of prior profile membership on subsequent behavior problems across two years. We expected that initial profile membership would statistically predict profile membership over the course of longitudinal assessments. This expected pattern is based on research showing that individual differences in persistent behavioral and emotional problems can be identified early in development (e.g., Jobs et al., 2019). Finally, our fourth aim was exploratory: to examine gender differences on latent profile membership at the initial assessment and over time (i.e., profile transitions) across the follow-up assessments.

## Method

### Sample

The present study used data from 2,341 preschool children from the general population who participate in the ELISA Project (*Estudio Longitudinal para una Infancia Saludable*; *Longitudinal Study for a Healthy Childhood*). The ELISA Project is an ongoing prospective study conducted in Galicia (North-western Spain) to analyze developmental pathways of child conduct, emotional behavior and psychosocial adjustment, starting from early childhood. Participating children (51.8% boys and 48.2% girls) were recruited from 72 schools (77.6% public, 17.1% charter, and 5.2% private) located in 27 urban, suburban and rural areas within Galicia. A large majority (98.2%) were Spanish, with only a 1% of participants reporting a different nationality, and with no recorded nationality for the remaining 0.8% of children. Further information about sample sociodemographic characteristics is in Table S1 in Supplemental Material. Data were collected from questionnaires completed by one caregiver per household (mostly mothers, but otherwise father or another primarily caregiver) at each time point: baseline (T1 = 2016–2017) as well as two subsequent annual assessment times (T2 = 2017–2018; T3 = 2018–2019). The adult rating child behavior was not necessary the same at each of the three time points. However, informant stability rates were high. The 76.63% of the sample had a stable informant during the follow-up period, which represents the 87.94% of the total cases with more than one-point of data. Participation rates were 95.21% in T1 (N = 2229), 84.49% in T2 (N = 1978) and 76.07% in T3 (N = 1781). ANOVA tests revealed that participants with longitudinally complete data (N = 1605; 49.8% boys and 50.2% girls) did not differ statistically from dropouts on anxiety and externalizing behaviors analyzed at each of the three time points. In addition, there was no age difference in dropout. All participants were included in the longitudinal analyses for examining developmental patterns of behavior.

### Measures

Behavioral problems were assessed via parents’ reports using the Children Behavior Checklist 1.5–5 (CBCL; Achenbach & Rescorla, 2000). In the third assessment timepoint, the subsequent version (for 6–18 yr olds) was used (Achenbach & Rescorla, 2001). Three CBCL DSM-Oriented Scales were analyzed: anxiety problems (ANX), oppositional defiant problems (OD), and attention deficit/hyperactivity problems (ADH). Item-responses were reported on a three-point Likert scale (0 = not true; 1 = somewhat or sometimes true; 2 = very true or often true). These scales have proven to be psychometrically sound in the assessment of emotional and behavioral problems within preschool and school aged children (Achenbach & Rescorla, 2000, 2001). As for reliability, in our study each subscale at each of the three assessments has demonstrated acceptable internal consistency (alpha coefficients ranging between 0.70 and 0.80). Evidence for validity of the scales has been provided in previous studies, with an adequate correspondence with CBCL syndrome scales (Achenbach et al., 2003) and mental health disorders assessed according to DSM diagnostic criteria (Ebesutani et al., 2010). Additionally, the intraclass correlation coefficient (ICC) at the school level for each subscale during each assessment points showed values of 0.017 and lower. This value indicates very low correlation of child problems within schools, well below that standard cutoff point of 0.05 for defining the presence of substantial clustering (Heck et al., 2014). Thus, we did not take nesting within schools into account in our analyses.

The Socioeconomic Status (SES) of the family was also included among the measures used in the present study (i.e., as a control variable). The family SES variable was indexed through variables about parents’ level of education and economic level of family (i.e., family income level and parents’ perception about financial solvency). Parents’ level of education was based on the average of the father’s and mother’s educational level reported on a six-point scale (1 = without basic studies; 6 = postgraduate studies). Family economic level was based on parents’ reports of family income rated on a four-point scale (1 = serious problems to make ends meet; 4 = well off). Family financial solvency to face daily overheads was rated on a five-point scale (1 = never worried; 5 = worried basically every day). A composite Family SES variable was computed by transforming these variables into z-scores and calculating the mean of these z-score variables. The same procedure to calculate a family SES variable can be found in a previous study that used data sample from the ELISA Project (López-Romero et al., 2019).

### Procedure

The ELISA Project procedure complies with all standard ethical guidelines, with the data collection for the current study having been approved by the Bioethics Committee of the Universidade de Santiago de Compostela and the Spanish Ministry of Economy and Competitiveness. Regarding recruitment, 126 heads of schools were contacted, of which 72 accepted the conditions and agreed for their institutions to take part in the study. Then families of around 5,300 preschoolers were contacted and invited to participate. An active consent form was finally filled out by families of 2,467 children (mean rate of 25–50% preschoolers per school). During an initial phase of presenting the project to families, heads of schools contributed to the handing out, collection and returning of the consent forms to the ELISA Project staff. During the data collection periods, teachers handled the delivery and collection of the ELISA Project questionnaires that were filled out by families. Additionally, teachers actively participated in the ELISA Project, completing a yearly brief questionnaire concerning each of the participating children from their classrooms (i.e., around 15 to 25 participants per classroom). This brief questionnaire for teachers was also shorter than the questionnaire for parents (e.g., teachers did not report the CBCL scales at T1).

The present study included data reported by parents who completed information on ADH, OD and ANX DSM-Oriented Scales of CBCL. These parents could choose to fill out the questionnaire in paper form or online. All the questionnaires were key-coded in order to guarantee confidentiality. Parents had one month to complete and return their questionnaires. Neither teachers nor parents received any economic compensation for their participation.

### Data Analysis

The study implemented latent variable mixture modelling with cross-sectional and longitudinal data. Firstly, *Latent Profile Analysis* (LPA) was applied at each time point to identify subgroups of children based on similar patterns, or *profiles*, of behavioral problems. LPA is a person-centered method employed to identify homogeneous latent profiles of individuals using a set of continuous variables as indicators. In this case, ADH, OD and ANX behavior problems scores were used as continuous indicators. Secondly, an autoregressive model designed as an extension of latent class/profile analysis for longitudinal data, the *Latent Transition Analysis* (LTA), was conducted to describe the stability and change of behavioral profiles over the two years following the first assessment.

All analyses were conducted using Mplus version 7.4 (Muthén & Muthén, 1998–2015). Data analysis was performed mainly following the steps suggested by Nylund (2007) to conduct LTA, which include the application of LPA.

#### Step 0: Study Descriptive Statistics

Sample descriptive statistics were explored for the observed variables.

#### Step 1: Study Measurement Model Alternatives for Each Time Point

Based on LPA, solutions from 2 to 6 profiles were examined for each of the three time points separately. Each analysis model was estimated using MLR for maximum likelihood estimation with robust standard errors. To avoid the convergence on a local solution (i.e., false maximum likelihood), we estimated the model using 3000 sets of random starts, 100 iterations, retaining 100 sets of starting values for final stage optimization. Best Log Likelihood (LL) for all models must be replicated more than twice on the final stage optimization for trustworthy solutions. To compare models and determine the empirically best solution regarding the number of profile groups, fit indices were examined: Akaike Information Criterion (AIC), Bayesian Information Criterion (BIC), and Sample-Adjusted Bayesian Information Criterion (SABIC); as well as the LL value. A lower value in these fit indices and a higher value in LL indicates a better-fitting model. We also revised the Bootstrapped Likelihood Ratio Test (BLRT) and the Lo-Mendel-Rubin Likelihood Radio Test (LMR-LRT) to compare a solution with a *K* number of profiles with a solution with *K*-1 profiles. Entropy was also considered, with values closer to 1 being representative of better classification (i.e., larger differences between profile groups). Another index to evaluate class separation was revised: the average posterior probabilities (AvePP). The AvePP is specific for latent profiles, with values higher than 0.70 indicating distinct and well-separated profiles.

While all these indices were inspected, some studies have claimed that BIC and BLRT values are the best empirical indicators for deciding on the number of groups or classes (Nylund, 2007). Hence our empirical criteria decision was based mainly on these indicators. Complementing the empirical results of the best fitting model, we based our final decision on interpretability and the theorical support for that solution based on previous empirical results. In terms of interpretability, each profile had to include at least 5% of the total participants, and also to differ in the magnitude and behavioral pattern of the symptoms when compared to other profiles.

Additionally, we tested whether the final profile solution (i.e., means, variances, class probabilities) was reproduced consistently across family SES groups (lower SES = 45.7% of sample; higher SES = 49.5% of sample; the 4.7% of the sample did not report SES and were excluded from this part of the analyses) and child age groups (3–4 year group = 59.1% of sample; 5–6 year group = 40.9% of sample). A sequence of multigroup LPA for each grouping variable was performed in order to estimate four different levels of measurement invariance across groups (from less to more restrictive model: number of classes, within-group means, within-group variability, and class probabilities). Following Morin et al. (2016), at least two indices of the model information criteria need to have the same or a lower value for the more restricted model when determining adequacy of the model.

#### Step 2: Examining Measurement Invariance Across Time

Next, in order to see whether profile measurement was invariant across time points (an assumption that must be tested, in order to compare children’s trajectories over time), we used LRTs (Likelihood Radio Tests) to compare a full measurement non-invariance (or freely estimated) model with; a) a full measurement invariance model, which assumed equal profile structures in all time points, and b) a partial measurement invariance model, which assumed equal profile structure for some time points. None of these models included autoregressive relations between latent variables.

#### Step 3: Explore Specification of the LTA without Covariates

We then ran a first-order LTA with two transition points (first, T1 to T2; second, T2 to T3), with freely estimating coefficients and no covariate. A first-order transition model addressed the stability and change allowed for a direct effect between T1 and T2, and between T2 and T3. As a result, information was represented in probability matrices, where diagonal values described stability, or individuals in time *T* who remain in the same profile assigned in *T*-1, as well as off-diagonal values which describe the movement between profiles, indicating individuals in time *T* who came from a different profile assigned in *T*-1. In this step, we also examined the stationarity assumption across the two transition point matrices and the applicability of a second-order transition effect between T1 and T3.

On the one hand, to test whether the transitions were stationary (i.e., equal) across time points, the freely estimated LTA (no covariates) was compared with its constrained analogue using the traditional LRT (Likelihood Ratio Test). No significant differences in LRT revealed an equal likelihood of change between profiles in the first transition point and the second, implying stationary patterns of transition across time. Then, to test the lasting effects between non-adjacent time points (T1 and T3), we compared first- and second-order LTA models using LRT. By using second order transitions we accounted for the developmental direct effect between T1 and T3, regardless of T2.

#### Step 4: Include Covariates in the LTA Model

Finally, in order to explore the differences between boys and girls among profiles and transitions across time points, we included gender as a covariate in a previously specified LTA, controlling also for the possible effects of family SES. Following Collins and Lanza (2010) instructions, we tested differences in profile membership at T1 and differences in profile transitions to T2 and T3 by including time-invariant covariates, which were allowed to have time-variant effects. Logistic regression coefficients (log-odd values) were obtained to calculate profile membership and transition probabilities matrices. The statistical effect and significance of those values addressed the gender differences to be placed within and move among behavioral profiles, compared to their probability of being in a “reference group”. As a reference group, we used the profile with lowest values in ADH, OD and ANX behaviors.

## Results

### Step 0: Study Descriptive Statistics and Tests of Mean Differences between Genders and Across Time

Means, standard deviations, and ranges of the studied variables at three time points are shown in Table 1. Overall, ADH was the most common behavior, followed by OD, and finally ANX. Boys were rated significantly higher on ADH in all time points and OD in T2 and T3, after applying a t-test comparison with girls. No gender differences were apparent for OD in T1 or ANX in any of the recorded time points. Moreover, for the entire sample as well as for the boy and girl subsamples, there were significant decreases in all behavior rates when compared to *T*-1; the exception was ANX, whose rate remained stable between T1 and T2 (see Table S2 in Supplemental Material).

**Table 1:** Descriptive statistics of variables in all assessment points considering the full sample, boys, and girls

	**T1**		**T2**		**T3**	
	Mean (SD)	Range	Mean (SD)	Range	Mean (SD)	Range
**ADH**						
Total	0.72 (0.45)	2.00	0.64 (0.44)	2.00	0.52 (0.42)	2.00
Boys	0.76 (0.47)	2.00	0.68 (0.46)	2.00	0.58 (0.45)	2.00
Girls	0.68 (0.43)	2.00	0.60 (0.42)	2.00	0.46 (0.37)	1.86
**OD**						
Total	0.60 (0.39)	2.00	0.54 (0.39)	2.00	0.45 (0.40)	2.00
Boys	0.61 (0.40)	2.00	0.55 (0.40)	2.00	0.49 (0.43)	2.00
Girls	0.58 (0.38)	2.00	0.52 (0.38)	2.00	0.41 (0.36)	1.60
**ANX**						
Total	0.52 (0.32)	1.70	0.51 (0.34)	1.80	0.38 (0.30)	1.67
Boys	0.50 (0.31)	1.70	0.51 (0.34)	1.80	0.39 (0.31)	1.67
Girls	0.53 (0.33)	1.80	0.52 (0.33)	1.80	0.37 (0.29)	1.56

*ADH* Attention Deficit/Hyperactive Problems, *OD* Oppositional Defiant Problems, *ANX* Anxiety Problems

### Step 1: Study Measurement Model Alternatives for Each Time Point

The best LL value was obtained and replicated for all the examined LPA models. Table S3 shows model fit information for solutions ranging from 2 to 6 profiles at each time point (see Supplemental Material). As profiles were added, the model fit was enhanced, with a continuous decrease in BIC, AIC and SABIC, and an increase in LL. Moreover, BLRT remained significant for all solutions, pointing towards an improvement in the loglikelihood difference distribution when further profiles were added to the model. Nevertheless, LMR-LRT distinguished a significantly better fit of four profiles when compared with three profiles (*p* < 0.001) and a non-significant improvement with the five profiles solution in T1 and T2. This result was not replicated in T3, where a four-profile solution did not provide a significant LMR-LRT when compared with the three profiles model (*p* = 0.068).

To gather more information about the best-fitting model at each timepoint we also considered the progression on BIC and LL as the number of profiles included in the model increased. Table S3 also shows that BIC decreased, and LL increased continuously, until they started to level out around the five profiles solution, for both T1 and T2. This same diminishing pattern for BIC did not appear in T3, where BIC and LL steadily decreased and increased, respectively, from the four- to six-profiles solutions. Additionally, the AvePP for each profile of each model solution (and in each one of the three timepoints) reached the cut-off point (0.70) for good class separation.

**Fig. 1:**
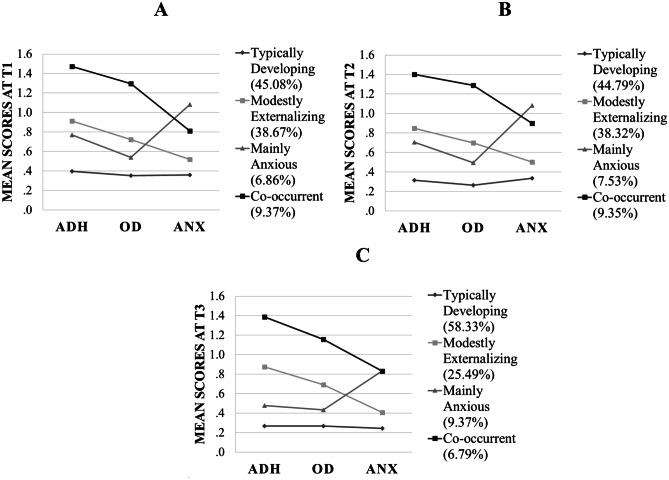
Attention Deficit/Hyperactivity (ADH), Oppositional Defiant (OD), and Anxious (ANX) behavior means for latent profile model at the three assessment times. Note: Fig. 1 shows combinations of higher versus lower values on ADH, OD and ANX mean scores, which conforms the four qualitative different behavioral profiles identified at T1 (A), T2 (B), and T3 (C): “typically developing” profile, with the lowest mean scores on ADH, OD and ANX; “modestly externalizing” profile, with a low mean score on ANX but medium on ADH and OD; “mainly anxious” profile, with the highest mean score on ANX but low scores on ADH and OD; “co-occurrent” profile, with medium scores on ANX for T1 and T2 (the highest in T3 along the “mainly anxious” profile) and the highest mean scores on ADH and OD at all-time points. Profile proportions are detailed, with the great majority of sampled children showing a “typically developing” or “modestly externalizing” profile, and minority showing a “co-occurrent” or “mainly anxious” profile

As the statistical fit indices did not indicate that there was one comparable solution for all three timepoints, we based our decision criteria on the interpretability, conceptual appropriateness, and parsimony of the model. At all time points, the two and three profiles solutions revealed behavioral patterns that differed only in terms of the magnitude of the symptoms. In contrast, more distinct information was provided by the models from the four-profiles solutions upwards. Specifically, the four-profiles model classified children into categories of “typically developing” and “modest externalizing” behavior, but also into two smaller categories of “mainly anxious” and “co-occurrent” behavior. The “mainly anxious” emerged in the four-profiles solution without splitting any of the previous three-profile groups. The more complicated five-profiles and six-profiles models also were descriptively rich but were discarded, because they added profile subgroups with fewer than 4 percent of the sampled children in a subgroup. We ultimately selected the four-profile model as the simplest and most interpretable solution for all three time points. This solution was further supported by former latent class/profile analysis studies; although those prior studies considered other variables that were not included in the present study (e.g., depression), they showed similar profile groups patterns (e.g., Basten et al., 2016). Percentages of participants and plotted means of the four profile groups are depicted for each time point in Fig. 1.

This four-profiles solution showed similar patterns across time points, but differences appeared in T3. In terms of the percentage of children classified into the distinct profiles, “typically developing” and “modestly externalizing” showed similar proportions in T1 and T2. In T3, however, a greater proportion of the children fit in the “typically developing” profile (58.33%), and fewer in the “modestly externalizing” group (25.49%). Additionally, we also noticed alterations in the size of the two less frequently assigned profiles across assessment points. In T1 and T2, a slightly higher percentage of children were classified into “co-occurrence” compared with “mainly anxious”, but these volumes were inverted in T3, with “co-occurrence” becoming the smaller sized profile group. Lastly, t-test comparisons (with Bonferroni correction) among profiles for each dimensional score (ADH, OD and ANX), at the three time points, revealed differences between the profiles in the assessed behaviors, with the exception of “co-occurrence” and “mainly anxious”, whose ANX mean score did not significantly differ in T3 (see Table S4 in Supplemental Material).

Additionally, sequences of multigroup LPA were conducted to test the consistency of the four-profile solution across family SES groups and child age groups at each of the three timepoints. Model fit information for testing measurement invariance across these grouping variables is shown in Table S5 and Table S6 (see Supplemental Material). A full restricted model, with fixed means, variances, and class probabilities across child age groups showed lower BIC and SABIC values than the less restrictive models. Thus, the four-profile solution was consistent across child age groups. In the case of family SES groups, the full restricted model failed to better fit to the data when compared with a previous less restrictive model. However, the full restrictive model showed lower BIC and SABIC values than the full unrestricted model (i.e., a model that assumed that the family SES groups are different). Additionally, a model that assumed equal means and variances across family SES groups showed lower BIC and SABIC values than a less restricted model and a full unrestricted model. Thus, the four-profile solution was also consistency across the family SES groups, in terms of within-group means, and within-group variability.

### Step 2: Examining Measurement Invariance

In agreement with the decreasing values of the observed scores in the behavior indicators (see Step 0), and confirming that there were variations in the profiles across time points, a full measurement non-invariance model obtained a better fit when compared with a full measurement invariance model (*X*^2^ *diff* (24) = 114.23, *p* < 0.001). Furthermore, to address the model fit discrepancies between the first two time points and the final one (see Step 1), the full measurement non-invariance was compared with a partial measurement invariance model that assumed equal profiles for T1 and T2. As a result, the varying profiles across time points were revealed to be better fitted than equal profiles for T1 and T2 (*X*^2^ *diff* (12) = 44.95, *p* < 0.001). Table S7 displays model fit information used to test the measurement invariance (see Supplemental Material).

### Step 3: Explore Specification of the Latent Transition Model without Covariates

Next, we compared LTA full measurement non-invariant models with different specifications to describe the stability and change among behavioral profiles across time points, exploring the assumptions of stationary probabilities and second-order effects in child transition patterns. Table 2 shows latent transition probabilities between T1 and T2, and between T2 and T3 based on a first-ordered and freely estimated LTA. Starting with the direct effect between T1 and T2, we observed that most of the children remained in the behavioral profile in which they were initially classified. Considering each profile size in T1, children assigned to “typically developing” showed the highest rate of stability, with 93.9% of them remaining in this profile in T2. The “mainly anxious” profile had a stability of 91.7%, with most of its “movers” transitioning to “typically developing” (6%) in T2. The stability in “modestly externalizing” was 87.2%, with a similar rate of change towards “typically developing” (7.6%), and “co-occurrent” (5.2%) in T2. The profile with the lowest stability (i.e., the most movers) was “co-occurrent”, with 83.2% of children remaining in this profile, and 15.9% transitioning towards “modestly externalizing”. Regarding transitions between T2 and T3, we observed similar probability rates of stability and change from T1 to T2 reported above, for the profiles “typically developing” and “mainly anxious”. Nevertheless, for “modestly externalizing” children, stability decreased to 76.7%, with higher rates of transition towards “typically developing” (17.6%) than “co-occurrent” (5.6%). Finally, 69.4% of children assigned to “co-occurrent” remained in this profile, with an increase (compared to transitions from T1 to T2) of its movers towards “modestly externalizing” (29.1%).

**Table 2:** First ordered transition probabilities of change among behavioral profiles considering their sizes across transition points

		**T2**	**Typically Developing**	**Modestly Externalizing**	**Mainly Anxious**	**Co-occurrent**
		**N**	1016	778	295	252
**T1**	**N**					
**Typically Developing**	1002		**0.939**	0.043	0.015	0.004
**Modestly Externalizing**	800		0.076	**0.872**	0.000	0.052
**Mainly Anxious**	300		0.060	0.000	**0.917**	0.023
**Co-occurrent**	239		0.000	0.159	0.010	**0.832**
		**T3**	**Typically Developing**	**Modestly Externalizing**	**Mainly Anxious**	**Co-occurrent**
		**N**	1097	724	287	233
**T2**	**N**					
**Typically Developing**	1016		**0.958**	0.017	0.022	0.003
**Modestly Externalizing**	778		0.176	**0.767**	0.000	0.056
**Mainly Anxious**	295		0.083	0.000	**0.898**	0.020
**Co-occurrent**	252		0.000	0.291	0.015	**0.694**

#### Stationarity

We tested the stationary assumption by comparing a freely estimated model with one that assumes equal transitions probabilities among profiles over time. Non-significant differences between these two models (*X*^2^ *diff* (12) = 10.79, *p* = 0.546) revealed that children generally followed a stationary pattern to move out of behavioral profiles over the assessed developmental period.

#### Second-Order Effects (i.e., Transitions from T1 to T3)

A comparison between first and second-ordered LTA models revealed a significant direct effect between non-adjacent time points (*X*^2^ *diff* (9) = 28.63, *p* = 0.001), implying that profile classification in T3 was related to the profile assigned in T1. In agreement with the results presented so far, behavioral profiles also showed high rates of stability in their lasting effects from T1 to T3 (see Table S8 in Supplemental Material). Specifically, 98% and 92.9% of children that initially belonged to “typically developing” and “mainly anxious” profiles respectively, were assigned to the same profile in T3, regardless of their position in T2. Moreover, “modestly externalizing” also showed a high stability between T1 and T3, with most movers shifting to “typically developing” (13.3%). Finally, even though “co-occurrent” was the least stable profile, more than a half of the children initially classified within this profile remained there (76.4%) from T1 to T3, while the rest transited towards “modestly externalizing” (23.1%). Overall, children initially classified as “co-occurrent” were 254.66 times more likely to end up in this profile at T3 than “typically developing” children; 18.63 times more than “modestly externalizing” children; and 44.94 times more than “mainly anxious” children.

### Step 4: Include Covariates in the LTA Model

We next included both gender and family SES as covariates in the first-order LTA model, with the purpose being to examine gender differences in latent profile membership and transitions after controlling for the effects of family SES. We found that gender was a significant predictor of transitions among latent profiles only towards the “co-occurrent” profile. Specifically, the logistic regression equations showed that boys were 4.51 times more likely than girls to transition from T1 to T2 (B = 1.51; *p* = 0.019) towards the “co-occurrent” profile, compared to the transitions they made to “typically developing” between T1 and T2, when controlling for family SES. As well, boys were 3.5 times more likely than girls to transition from T2 to T3 (B = 1.25, *p* = 0.006) towards the “co-occurrent” profile, compared to the transitions they made to “typically developing” between T2 and T3, when controlling for family SES. There were no other significant gender differences (see Table S9 in Supplemental Material).

In accordance with these logistic regression results, a generally similar pattern of stability and change emerged among behavioral profiles for boys and girls (see Table S10 in Supplemental Material). As we noted above, gender differences in child transition probabilities were found in the direction of the “co-occurrent” profile, from T1 to T2 and from T2 to T3. At the first transition point, 86.2% of boys versus 78.5% of girls remained in this profile, 13.2% of boys versus 18.7% of girls moved into it from “modestly externalizing”, and 0.6% of boys versus 2.8% of girls moved into it from “mainly anxiety”. At the second transition point, 74% of boys versus 52.9% of girls remained in the “co-occurrent” profile, 25% of boys versus 44.8% of girls moved into it from “modestly externalizing”, and 1% of boys versus 2.3% of girls moved into it from “mainly anxiety”.

## Discussion

The overall aim of the current study was to examine the development of co-occurrent anxiety and externalizing problems during early childhood, from a person-centered perspective. As a first objective, we sought to identify profiles based on combinations of these problems. Specifically, we explored the extent to which ANX, OD and ADH behaviors tend to combine in shaping co-occurring versus differentiated behavioral profiles. Our findings suggest that anxiety and externalizing problems during early childhood are distributed around four quantitatively and qualitatively different profiles, which we named as: “typically developing”, “mainly anxious”, “modestly externalizing”, and “co-occurrent”. A similar type of a four-profile solution was found in former studies which explored the association between internalizing and externalizing problems during childhood using the same or similar methods to those we used (Basten et al., 2016; McElroy et al., 2017; Morales et al., 2021; Willner et al., 2016).

Regarding profile sizes, the great majority of children at T1 were classified in the “typically developing” and “modestly externalizing” profiles, with some size changes over the subsequent two follow-ups. Members of the “typically developing” profile increased progressively over time, whereas those in “modestly externalizing” decreased. These results are consistent with the idea that behavioral problems show a tendency to diminish across the preschool years (D’Souza et al., 2019). Even so, several studies suggest that there are more externalizing than internalizing problems during early childhood (Fanti & Henrich, 2010). Consistent with that notion, more children exhibited a “modestly externalizing” profile than “mainly anxious” and “co-occurrent” ones, across the three assessment points. We also found a slight increase in children presenting a “mainly anxious” profile at T3 with respect to T1, which might be related to a gradual elevation of anxiety self-reporting as children gain cognitive skills to better express their feelings to their parents and caregivers (Gilliom & Shaw, 2004). Regarding the “co-occurrent” profile, the proportion displayed (between 6–10% depending on time point) was comparable to comorbidity rates of anxiety and externalizing disorders in preschoolers reported by Wichstrøm et al. (2012).

With respect to behavior intensity, the “typically developing” profile was characterized by the lowest rates on externalizing and anxiety behaviors. This implies that the other profiles showed both problems to a certain degree, as was expected due to the low specificity of early developmental problems (Finsaas et al., 2018; Jobs et al., 2019). In addition, the “co-occurrent” profile group had children who showed a pattern of higher levels of problem behaviors, as we hypothesized in light of previous work (e.g., Basten et al., 2013, 2016; McElroy et al., 2017; Willner et al., 2016). Research suggests that co-occurrent behavioral patterns based on externalizing and anxiety problems represent a high-risk profile (Yoo et al., 2009). It should be noted that the “co-occurrent” profile identified in the current study showed more externalizing problems than any other, and more anxiety than the “modestly externalizing” and “typically developing” profiles, but not more than “mainly anxious”. However, after two years of follow-up, the anxiety level in the “co-occurrent” profile was equal to that of “mainly anxious”, depicting a similar pattern to Willner et al. (2016) in their at-risk sample of young children. Taken together these findings complement research which relates the co-occurrence of anxiety and attention deficit/hyperactivity disorders with the highest levels of behavioral problems in childhood (Danforth et al., 2019; Humphreys et al., 2012). Such research was itself based on studies suggesting that anxiety could aggravate the behavioral inhibitory response in children with ADHD (Sørensen et al., 2011), thus contributing to the presence of more externalizing problems.

The second aim of the current study was to analyze the change and stability of the four behavioral profiles over two years. Overall, our findings revealed more stability than change in terms of profile membership (like Willner et al., 2016). Thus, although some children may experience a normative-diminished pattern of behavior difficulties during early development, there is also persistent problems from early childhood for a subgroup of children (Briggs-Gowan et al., 2006; Jobs et al., 2019). These persistent behavior problems continue over time in their presentation and severity (Finsaas et al., 2018). Accordingly, our results showed that the behavioral profiles identified in a community sample of preschoolers tended to persist over two years. Thus, we suggest that identifying a subgroup of preschoolers with a certain behavioral profile, instead of focusing on the expression of a single problem dimension at a time (i.e., variable-centered analysis), will reduce the challenge of predicting the courses of risk behavioral trajectories which have started early in development.

It is worth stressing that each behavioral profile showed a particular pattern of change and stability over time. Thus, children showing a “typically developing” profile followed the most stable behavioral course. Moreover, a substantial amount of transitioning from other profiles ended up in the “typically developing” profile by T3. As previously mentioned in the Introduction, it was expected that young children (in contrast to older children and adolescents, for instance) would be more likely to minimize behavioral problems over time as part of normative development, in line with the concept of discontinuity in developmental psychopathology. Conversely, the “co-occurrent” profile was the least stable one over the follow-up period. In agreement with the *differentiation* hypothesis, preschoolers who exhibited a “co-occurrent” profile in T1 tended to (primarily) experienced a reduction in their anxiety behavior over time, finding better adjustment in the “modestly externalizing” profile. Nevertheless, most of the children assigned to the “co-occurrent” profile showed a greater probability of remaining stable than of moving to another profile with a less intense expression of behavioral problems. Further research should examine whether an early stability of a co-occurrent profile responds to the presence of factors and processes that are common for anxiety and externalizing behaviors due to shared or overlapping common risk factors. The presence of those factors and processes might confer on the child a special vulnerability to experience these problems together.

We also found some child transitioning from the “modestly externalizing” profile towards the “co-occurrent” one, although this was less common compared to the transition described above. This might be because there is a small subgroup of preschoolers whose behavioral profiles become worse over the course of early childhood. Since these transitions were more marked from T2 to T3, we hypothesize that future follow-ups of this sample (as they develop across middle childhood and into early adolescence) would show a strengthening of this phenomenon, providing a basis for testing the accumulation hypothesis. This is in line with the idea that externalizing problems might be related to further internalizing problems during developmental moments of more complex and intimate interactions with peers, and because of a greater cognitive capacity and ability of the child to self-evaluate (Oland & Shaw, 2005). Consequently, direct and indirect effects between these problems might better explain co-occurrence during transitional periods, such as the transition to elementary school or to adolescence. It also is important, when testing the accumulation hypothesis in an early stage of development, to examine the relationships among different forms of these problem behaviors. Certain subtypes of anxious behavior become more prevalent after early childhood (e.g., social anxiety) (Steinsbekk et al., 2021); considering them might reveal distinctions among profiles. This could help clarify the mixed results regarding the role of anxiety (risk vs. protective effect) on externalizing problems (Drabick et al., 2010) that remains an open question in the field (Danforth et al., 2019; Murray et al., 2018).

Regarding the third aim of testing the lasting effects of early membership profile status on future behavior, our study revealed that progress through profiles showed consistent patterns of stability and change throughout assessments over time (i.e., the assumption of stationarity). Moreover, the initial assignment to a certain profile group had lasting effects on the profile exhibited two year later. These results call for further explorations of early factors linked with these behavioral profiles based on anxiety and externalizing problems at the preschool age, since their detection at even earlier ages would allow for the development of tailored intervention tools at younger ages before stable profiles of behavioral and emotional problems have solidified. Furthermore, our conclusion that changes from differentiated to co-occurring problems should be unusual during early childhood also raises the question: during early childhood, does the emergence of co-occurrence reflect causes from common factors or the accumulation of the problems across dimensions (Willner et al., 2016)? Longitudinal studies with longer follow-up periods could explore the possibility that the explanatory mechanisms of co-occurrence vary throughout a child’s development. Thus, the high comorbidity rates observed later, in middle childhood for instance, might be a result of the increasing levels of reported anxiety during this period (Marmorstein, 2007; Russo & Beidel, 1994), but also perhaps due to the accumulation of the processes with which the co-occurrence with externalizing problems originated.

Regarding our fourth and final aim, we explored gender differences both in terms of belonging to a behavioral profile at pre-school, and as changing towards a certain profile over the course of follow-ups. Findings suggest similarities for boys and girls in membership of the “modestly externalizing” and the “mainly anxious” profiles; more boys than girls, though, were likely to transit towards the “co-occurrent” profile at follow-up points. Taking into account that the “co-occurrent” profile was characterized as displaying the highest rates of externalizing behavior, our results are consistent with research that indicates significantly more externalizing problems (Costello et al., 2005) and significantly more relation between externalizing and internalizing problems in boys than girls (Basten et al., 2013, 2016; Danzig et al., 2013; Marmorstein, 2007). This preponderance of boys in the group with more intense behavioral problems during early childhood might change over development (Rutter et al., 2003), thus our findings support the importance of considering gender when examining the temporal interplay of anxiety and externalizing symptoms over time.

However, our results must be viewed in light of certain limitations. First, data analyses were based on behavioral problems reported by only one caregiver, which were mainly mothers. Moreover, the caregiver who reported the child behaviors was not necessarily the same person across assessments, although informant stability rates were high. In order to control for the effect of multiple informants in our study, preliminary LTA were conducted using only mothers’ behavioral ratings (i.e., using the 80.94% of the sample), and the results were very similar to those reported. Further research should integrate ratings of multiple informants (e.g., fathers and teachers) as a means of enhancing measurement methods, and for estimating informant and home vs. school context effects (Alexander et al., 2017; Herman et al., 2018). Second, we selected a profile solution that was congruent with prior studies on co-occurrent profiles based on internalizing and externalizing problems during early childhood development (e.g., Basten et al., 2016; Willner et al., 2016). Subsequent work might well contemplate the inclusion of specific predictor variables in order to better describe these profiles, such as child levels of self-regulation (Ip et al., 2019) and social problems (Shi et al., 2020), as well as parental control and affect (Wiggins et al., 2015; Zubizarreta et al., 2019). This would be a way of examining the shared risk processes of anxiety and externalizing problems on the development of their co-occurrence, an issue which remains as an open question. Third, we did not account for different sub-dimensions of anxiety, oppositional defiant or attentional deficit/hyperactive problems, which would lead to an even better understanding of the specific relationships between the problems in question. As well, these problems subdimensions can be included into latent profile and transition analyses in order to increase the number of observed variables in the model. Adding theory-related variables with potential for delineating profiles into these analyses might enhance model estimation (Wurpts & Geiser, 2014). In this study, although the measurement model was based only on three observed variables, the fact that these variables were continuous and performed in the context of a large sample size probably may compensate the estimation bias related with the use of few indicators. Fourth, results revealed a structure of behavioral profile stability and change that might be common for the broad age range (3–6 years) in the current study. As already noted, previous studies applying latent profile models on externalizing and internalizing problems during early childhood obtained a similar configuration of the profiles. Moreover, the four-profile solution shown in the present study has been statistically replicated across child age groups along assessment points. Additional support for the structural and dispersion similarity of these profiles was obtained after controlling for family SES. Notwithstanding, conducting LTA by age-groups might reveal more detailed observations of the transitioning among profiles across time points. Thus, conducting LTA for each year of age is needed to more rigorously test and further validate these findings. Lastly, this study accounted for the possible deviations of behaviors from a normative group in our sample, assuming problems in a continuum from less to greater intensity. Therefore, profile labels were set accordingly with the results of a comparative test, rather than the absolute levels of behaviors. Thus, for example, the anxiety levels in the co-occurrent profiles were not high in absolute terms, but still indicated a distinctive characteristic of this profile across assessment points. Since our sample is community-based, research should also examine clinical samples (e.g., referred, treated) in order to test the generalizability of these findings.

To the best of our knowledge, this is the first study to examine the early development of behavioral patterns of co-occurrent anxiety and externalizing problems by employing LTA. The study results show that behavioral profiles at preschool ages predict child behavioral profiles two years later. In other words, children who maintain a stable pattern of difficulties were identified during the preschool years. This provides evidence that prevention and intervention efforts should be directed towards preschoolers (something the field already knew), but our findings also provide support for the need to focus on overall child behavioral patterns rather than on single problem behavior dimensions—such profile groups are much more informative about a child´s particular characteristics. Specifically, our results suggest that an early stable co-occurrent profile of anxiety and externalizing problems can be identified during preschool and that this behavioral pattern might be associated with a high-risk profile. This merits particular attention, given that early preventive interventions for this configuration of co-occurring problems are scarce in the research literature. Furthermore, there is a need to identify even earlier developmental predictors of co-occurrent anxiety and externalizing problems in early childhood. Further research would complement and extend our findings, by identifying individual and family predictors of profile membership and transitioning during early childhood. Additionally, studies that extend their assessment to late childhood or adolescence should consider measuring the various forms of anxiety and externalizing behavior, as both the specification of and associations between these specific types of behavioral problems become more complex as development progresses. Doing so will more accurately and completely account for the broad range of children’s vulnerability and likely responsiveness to developmentally appropriate prevention and intervention approaches.

## Supplementary Information

Below is the link to the electronic supplementary material.

Supplementary file1 (DOCX 72 KB)

## Data Availability

The datasets analyzed and the code used during the current study are available from the corresponding author on request.
