# Supplementary material for: The Development of Co-Occurrent Anxiety and Externalizing Problems from Early Childhood: a Latent Transition Analysis Approach
Source: Res Child Adolesc Psychopathol. 2021 Sep 9;50(4):505–19. doi: 10.1007/s10802-021-00865-2 (PMC8940780; doi:10.1007/s10802-021-00865-2)
Supplement: Supplementary file 1 — Supplementary file1 (DOCX 72 KB) [file 10802_2021_865_MOESM1_ESM.docx]

**SUPPLEMENTAL MATERIAL**

**The development of co-occurrent anxiety and externalizing problems from early childhood: A Latent Transition Analysis approach**

**Table S1**

*Participants Sociodemographic Characteristics*

| **Child gender** (N=2341) | Girls | 48.2% |
| --- | --- | --- |
|  | Boys | 51.8% |
| **Mean age** (SD) | T1 (N=2229) | 4.24 (.90) |
|  | T2 (N=1978) | 5.34 (.92) |
|  | T3 (N=1781) | 6.31 (.92) |
| **Survey respondent in T1** | Mother | 87.2% |
|  | Father | 12.5% |
|  | Other | .3% |
| **Survey respondent in T2** | Mother | 74.05% |
|  | Father | 10.4% |
|  | Other | .3% |
| **Survey respondent in T3** | Mother | 67% |
|  | Father | 9.4% |
|  | Other | .4% |
| **Mother’s mean age at childbirth** |  | 33.75 |
| **Father’s mean age at childbirth** |  | 35.60 |
| **Maternal education in T1** | Compulsory | 14.2% |
|  | Post compulsory | 38.2% |
|  | Higher | 47.5% |
| **Paternal education in T1** | Compulsory | 30.1% |
|  | Post compulsory | 38.7% |
|  | Higher | 31.2% |
| **Perception of family income in T1** | Extremely low | 1.2% |
|  | Low | 6.2% |
|  | Medium | 48.3% |
|  | High | 44.3% |

**Note**. Compulsory: Primary and low secondary mandatory education; Post compulsory: Upper secondary and professional courses; Higher: graduates, masters and doctorates.

**Table S2**

*Behavioral rate differences across assessment points* *considering the full sample, boys, and girls*

|  | **T1 – T2** | | **T2 – T3** | |
| --- | --- | --- | --- | --- |
|  | Mean (SD) | *t* (df) | Mean (SD) | *t* (df) |
| **ADH** |  |  |  |  |
| Total | .07 (.35) | 9.15 (1886)** | .13 (.35) | 14.63 (1662)** |
| Boys | .08 (.36) | 6.57 (962)** | .11 (.38) | 8.77 (831)** |
| Girls | .08 (.35) | 6.36 (923)** | .14 (.34) | 12.11 (830)** |
| **OD** |  |  |  |  |
| Total | .06 (.34) | 7.31 (1885)** | .09 (.34) | 10.57 (1662)** |
| Boys | .05 (.33) | 4.99 (962)** | .07 (.35) | 4.71 (831)** |
| Girls | .06 (.34) | 5.38 (922)** | .11 (.33) | 9.33 (830)** |
| **ANX** |  |  |  |  |
| Total | .01 (.28) | .79 (1886) | .13 (.23) | 18.06 (1662)** |
| Boys | <.01 (.28) | .12 (962) | .11 (.29) | 11.31 (831)** |
| Girls | .01 (.29) | .93 (923) | .14 (.28) | 14.30 (830)** |

**Note**. ADH= Attention Deficit/Hyperactive Problems; OD= Oppositional Defiant Problems; ANX= Anxiety Problems.

** *p* < .001

**Table S3**

Model fit information for Latent Profile Analysis (LPA) solutions ranging from 2 to 6 profiles at each time point

|  | **AIC** | **BIC** | **SABIC** | **LL** | **2LLDiff** | **LMR-LRT** | ***p* BLRT** | **ENT** | **SPS (%)** |
| --- | --- | --- | --- | --- | --- | --- | --- | --- | --- |
| **Child Behavior LPA T1** |  |  |  |  |  |  |  |  |  |
| 2 Profiles | 5277.81 | 5334.90 | 5303.13 | -2628.90 | 1045.39 | 1012.56** | .000 | .75 | 23.50% |
| 3 Profiles | 4987.48 | 5067.41 | 5022.93 | -2479.74 | 298.33 | 288.96** | .000 | .69 | 9.87% |
| 4 Profiles | 4874.47 | 4977.24 | 4920.05 | -2419.23 | 121.01 | 117.21** | .000 | .70 | 6.86% |
| 5 Profiles | 4814.70 | 4940.31 | 4870.41 | -2385.35 | 67.76 | 65.64 (*p*=.206) | .000 | .72 | 3.37% |
| 6 Profiles | 4778.20 | 4926.64 | 4844.03 | -2363.10 | 44.51 | 43.11 (*p*=.319) | .000 | .72 | 2.06% |
| **Child Behavior LPA T2** |  |  |  |  |  |  |  |  |  |
| 2 Profiles | 4724.06 | 4779.96 | 4748.19 | -2352.03 | 1050.36 | 1016.87** | .000 | .71 | 28.16% |
| 3 Profiles | 4283.09 | 4361.35 | 4316.87 | -2127.54 | 448.97 | 434.66** | .000 | .75 | 9.40% |
| 4 Profiles | 4135.91 | 4236.53 | 4179.34 | -2049.96 | 155.18 | 150.23** | .000 | .76 | 7.53% |
| 5 Profiles | 4077.41 | 4200.39 | 4130.49 | -2016.71 | 66.50 | 64.38 (*p*=.532) | .000 | .77 | 3.99% |
| 6 Profiles | 4044.72 | 4190.05 | 4107.45 | -1996.36 | 40.69 | 39.40 (*p*=.394) | .000 | .80 | 3.79% |
| **Child Behavior LPA T3** |  |  |  |  |  |  |  |  |  |
| 2 Profiles | 3587.89 | 3642.74 | 3610.97 | -1783.95 | 1057.14 | 1022.97** | .000 | .80 | 23.52% |
| 3 Profiles | 3325.58 | 3402.37 | 3357.89 | -1648.79 | 270.31 | 261.58** | .000 | .81 | 6.73% |
| 4 Profiles | 3221.49 | 3320.22 | 3263.03 | -1592.74 | 112.09 | 108.47 (*p*=.068) | .000 | .78 | 6.79% |
| 5 Profiles | 3132.46 | 3253.13 | 3183.23 | -1544.23 | 97.03 | 93.98 (*p*=.321) | .000 | .80 | 3.59% |
| 6 Profiles | 3041.82 | 3184.43 | 3101.83 | -1494.91 | 98.64 | 95.45** | .000 | .80 | 3.03% |

**Note**. AIC= Akaike Information Criterion; BIC= Bayesian Information Criterion; SABIC= Sample-Adjusted Bayesian Information Criterion; LL= Log Likelihood; 2LLDiff= 2 Times the Loglikelihood Difference; LMR-LRT= Lo-Mendel-Rubin Likelihood Radio Test; *p* BLRT= *p* value of Bootstrapped Likelihood Ratio Test; ENT= Entropy; SPS= Smaller Profile Size.

** *p* < .001

**Table S4**

*Difference among behavioral profiles on CBCL dimensional scores at each time point, applicating Bonferroni test for multiple comparison*

| **CBCL** | **(I) Profile** | **(J) Profile** | **Mean Diff (I-J) (SD)** | | |
| --- | --- | --- | --- | --- | --- |
|  |  |  | **T1** | **T2** | **T3** |
| **ADH** | **Modestly Externalizing** | Typically Developing | .58 (.01)** | .57 (.01)** | .64 (.01)** |
|  |  | Mainly Anxious | .18 (.02)** | .17 (.02)** | .43 (.02)** |
|  |  | Co-occurrent | -.56 (.02)** | -.54 (.02)** | -.51 (.02)** |
|  | **Mainly Anxious** | Typically Developing | .40 (.02)** | .40 (.02)** | .21 (.02)** |
|  |  | Co-occurrent | -.74 (.03)** | -.72 (.03)** | -.94 (.03)** |
|  | **Co-occurrent** | Typically Developing | 1.14 (.02)** | 1.11 (.02)** | 1.15 (.02)** |
|  |  |  |  |  |  |
| **OD** | **Modestly Externalizing** | Typically Developing | .41 (.01)** | .46 (.01)** | .44 (.02)** |
|  |  | Mainly Anxious | .23 (.02)** | .24 (.02)** | .30 (.03)** |
|  |  | Co-occurrent | -.57 (.02)** | -.59 (.02)** | -.45 (.03)** |
|  | **Mainly Anxious** | Typically Developing | .19 (.02)** | .21 (.02)** | .15 (.02)** |
|  |  | Co-occurrent | -.81 (.03)** | -.83 (.03)** | -.75 (.04)** |
|  | **Co-occurrent** | Typically Developing | 1.0 (.02)** | 1.05 (.02)** | .90 (.03)** |
|  |  |  |  |  |  |
| **ANX** | **Modestly Externalizing** | Typically Developing | .17 (.01)** | .17 (.01)** | .17 (.01)** |
|  |  | Mainly Anxious | -.65 (.02)** | -.66 (.02)** | -.48 (.02)** |
|  |  | Co-occurrent | -.31 (.02)** | -.40 (.02)** | -.45 (.02)** |
|  | **Mainly Anxious** | Typically Developing | .82 (.02)** | .83 (.02)** | .65 (.02)** |
|  |  | Co-occurrent | .34 (.02)** | .26 (.03)** | -.03 (.02) |
|  | **Co-occurrent** | Typically Developing | .47 (.02)** | .58 (.02)** | .61 (.02)** |

**Note**. ADH= Attention Deficit/Hyperactive Problems; OD= Oppositional Defiant Problems; ANX= Anxiety Problems; Mean Diff (I-J)= Mean difference among (I) Profile and (J) Profile.

** *p* < .001

**Table S5**

Model fit information for sequences of Multigroup Latent Profile Analysis conducted to test replicability of the 4-profile solution across children age subgroups

|  | **#Par** | **LL** | **SCF** | **AIC** | **BIC** | **SABIC** | **ENT** |
| --- | --- | --- | --- | --- | --- | --- | --- |
| **T1** |  |  |  |  |  |  |  |
| **Number of classes** | 37 | -3931.93 | 1.29 | 7937.86 | 8149.11 | 8031.55 | .79 |
| **Within-group means** | 25 | -3918.60 | 1.22 | 7887.19 | 8029.92 | 7950.50 | .80 |
| **Within-group variability** | 22 | -3919.93 | 1.26 | 7883.86 | 8009.46 | 7939.57 | .80 |
| **Class probabilities** | 19 | -3923.50 | 1.27 | 7884.99 | 7993.46 | 7933.10 | .80 |
| **T2** |  |  |  |  |  |  |  |
| **Number of classes** | 37 | -3369.04 | 1.29 | 6812.09 | 7018.91 | 6901.36 | .85 |
| **Within-group means** | 25 | -3377.49 | 1.20 | 6804.98 | 6944.73 | 6865.30 | .84 |
| **Within-group variability** | 22 | -3379.30 | 1.21 | 6802.60 | 6925.58 | 6855.69 | .84 |
| **Class probabilities** | 19 | -3380.28 | 1.23 | 6798.55 | 6904.76 | 6844.39 | .84 |
| **T3** |  |  |  |  |  |  |  |
| **Number of classes** | 37 | -2773.85 | 1.39 | 5621.69 | 5824.63 | 5707.09 | .88 |
| **Within-group means** | 25 | -2796.22 | 1.47 | 5642.44 | 5779.56 | 5700.14 | .85 |
| **Within-group variability** | 22 | -2797.99 | 1.48 | 5639.98 | 5760.65 | 5690.76 | .85 |
| **Class probabilities** | 19 | -2798.52 | 1.54 | 5635.03 | 5739.25 | 5678.88 | .85 |

**Note**. #Par= Number of Free Parameters; LL= Log Likelihood; SCF= Scaling Correction Factor for MLR; AIC= Akaike Information Criterion; BIC= Bayesian Information Criterion; SABIC= Sample-Adjusted Bayesian Information Criterion; ENT= Entropy.

**Table S6**

Model fit information for sequences of Multigroup Latent Profile Analysis conducted to test replicability of the 4-profile solution across family SES subgroups

| **Levels of measurement invariance** | **#Par** | **LL** | **SCF** | **AIC** | **BIC** | **SABIC** | **ENT** |
| --- | --- | --- | --- | --- | --- | --- | --- |
| **T1** |  |  |  |  |  |  |  |
| **Number of classes** | 37 | -3893.38 | 1.33 | 7860.77 | 8071.83 | 7954.27 | .80 |
| **Within-group means** | 25 | -3907.04 | 1.23 | 7864.08 | 8006.69 | 7927.26 | .80 |
| **Within-group variability** | 22 | -3907.45 | 1.26 | 7858.89 | 7984.39 | 7914.49 | .80 |
| **Class probabilities** | 19 | -3941.73 | 1.28 | 7921.46 | 8029.84 | 7969.47 | .80 |
| **T2** |  |  |  |  |  |  |  |
| **Number of classes** | 37 | -3208.06 | 1.27 | 6490.12 | 6695.24 | 6577.69 | .84 |
| **Within-group means** | 25 | -3222.75 | 1.21 | 6495.50 | 6634.09 | 6554.67 | .84 |
| **Within-group variability** | 22 | -3223.07 | 1.22 | 6490.15 | 6612.11 | 6542.22 | .84 |
| **Class probabilities** | 19 | -3241.46 | 1.23 | 6520.93 | 6626.26 | 6565.89 | .84 |
| **T3** |  |  |  |  |  |  |  |
| **Number of classes** | 37 | -2654.80 | 1.49 | 5383.60 | 5584.93 | 5467.38 | .86 |
| **Within-group means** | 25 | -2671.55 | 1.44 | 5393.09 | 5529.12 | 5449.70 | .85 |
| **Within-group variability** | 22 | -2672.33 | 1.49 | 5388.65 | 5508.36 | 5438.47 | .85 |
| **Class probabilities** | 19 | -2686.64 | 1.56 | 5411.27 | 5514.66 | 5454.30 | .86 |

**Note**. #Par= Number of Free Parameters; LL= Log Likelihood; SCF= Scaling Correction Factor for MLR; AIC= Akaike Information Criterion; BIC= Bayesian Information Criterion; SABIC= Sample-Adjusted Bayesian Information Criterion; ENT= Entropy.

**Table S7**

Model fit information for measurement invariance

|  | **#Par** | **LL** | **SCF** | **AIC** | **BIC** | **SABIC** | **ENT** |
| --- | --- | --- | --- | --- | --- | --- | --- |
| **Full invariance** | 30 | -6135.59 | 1.43 | 12331.17 | 12503.92 | 12408.61 | .68 |
| **T1-T2 invariance** | 42 | -6086.84 | 1.44 | 12257.67 | 12499.52 | 12366.08 | .66 |
| **Full non-invariance** | 54 | -6061.93 | 1.37 | 12231.87 | 12542.82 | 12371.25 | .66 |

**Note**. #Par= Number of Free Parameters; LL= Log Likelihood; SCF= Scaling Correction Factor for MLR; AIC= Akaike Information Criterion; BIC= Bayesian Information Criterion; SABIC= Sample-Adjusted Bayesian Information Criterion; ENT= Entropy.

**Table S8**

Second ordered transition probabilities of change among behavioral profiles considering their sizes between T1 and T3, collapsing T2

|  |  | **T3** | **Typically Developing** | **Modestly Externalizing** | **Mainly Anxious** | **Co-occurrent** |
| --- | --- | --- | --- | --- | --- | --- |
|  |  | **N** | 1109 | 726 | 280 | 226 |
| **T1** | **N** |  |  |  |  |  |
| **Typically Developing** | 1006 |  | **.980** | .011 | .006 | .003 |
| **Modestly Externalizing** | 798 |  | .133 | **.826** | .000 | .041 |
| **Mainly Anxious** | 295 |  | .054 | .000 | **.929** | .017 |
| **Co-occurrent** | 242 |  | .004 | .231 | .000 | **.764** |

**Table S9**

Logistic regression estimates and odds ratio of latent profile membership at T1 and across time transitions among latent profiles considering gender (boys = 1, girls = 0) as a covariate on a first-order LTA

|  | **Estimate** | **S.E.** | **Est./S.E.** | **Odd ratio** |
| --- | --- | --- | --- | --- |
| **Profile membership T1** |  |  |  |  |
| Modestly Externalizing | .13 | .13 | .97 | 1.14 |
| Mainly Anxious | -.29 | .17 | -1.76 | .75 |
| Co-occurrent | .32 | .17 | 1.86 | 1.38 |
| **Transitions T1 to T2** |  |  |  |  |
| Modestly Externalizing | 1.07 | .56 | 1.91 | 2.91 |
| Mainly Anxious | -.12 | .72 | -.17 | .89 |
| Co-occurrent | **1.51*** | **.64** | **2.35** | **4.51** |
| **Transitions T2 to T3** |  |  |  |  |
| Modestly Externalizing | .33 | .33 | 1.00 | 1.40 |
| Mainly Anxious | .08 | .89 | .09 | 1.08 |
| Co-occurrent | **1.25*** | **.46** | **2.75** | **3.50** |

**Note**. “Typically Developing” profile as reference/comparison group. The model included family SES as control variable (not shown here).

* *p* < .05

**Table S10**

Boys/girls latent profile membership at T1 and transition probabilities across time points based on a first-order LTA

|  | **Typically Developing** | **Modestly Externalizing** | **Mainly Anxious** | **Co-occurrent** |
| --- | --- | --- | --- | --- |
| Profile Membership T1 | | | | |
|  | .413/.428 | .363/.331 | .114/.158 | .110/.082 |
| Transitions T1 (rows) to T2 (columns) | | | | |
| **Typically Developing** | **.915/.961** | .070/.025 | .011/.013 | .004/0.001 |
| **Modestly Externalizing** | .039/.107 | **.895/.852** | .000/.000 | .067/.041 |
| **Mainly Anxious** | .057/.053 | .000/.000 | **.906/.939** | .037/.008 |
| **Co-occurrent** | .000/.000 | .132/.187 | .006/.028 | **.862/.785** |
|  |  | |  | |
| Transitions T2 (rows) to T3 (columns) | | | | |
| **Typically Developing** | **.963/.970** | .023/.017 | .014/.013 | .000/.000 |
| **Modestly Externalizing** | .158/.216 | **.770/.756** | .000/.000 | .072/.028 |
| **Mainly Anxious** | .068/.075 | .000/.000 | **.902/.916** | .030/.009 |
| **Co-occurrent** | .000/.000 | .250/.448 | .010/.023 | **.740/.529** |

Mplus Syntax for Estimating LPA Models (**Step 1**)

TITLE: LPA 4-profile solution at T1

DATA: FILE IS lta.dat;

VARIABLE: NAMES ARE

case gender ses

f1adhd f1odd f1ans

f2adhd f2odd f2ans

f3adhd f3odd f3ans;

USEVARIABLES

f1adhd f1odd f1ans;

MISSING = ALL (999.00);

CLASSES c(4);

IDVARIABLE = case;

SAVEDATA: FILE is 4-lpa-t1.dat;

SAVE = CPROB;

ANALYSIS:

ESTIMATOR = MLR;

TYPE = MIXTURE;

STARTS = 3000 100;

STITERATIONS = 100;

PLOT: TYPE = PLOT3;

SERIES IS f1adhd f1odd f1ans (*);

OUTPUT:

SAMPSTAT STDYX TECH1 TECH7 TECH11 TECH14;

Mplus Syntax for Examining Measurement Invariance (**Step 2**)

TITLE: Full measurement invariance model

DATA: FILE IS lta.dat;

VARIABLE: NAMES ARE

case gender ses

f1adhd f1odd f1ans

f2adhd f2odd f2ans

f3adhd f3odd f3ans;

USEVARIABLES

f1adhd f1odd f1ans

f2adhd f2odd f2ans

f3adhd f3odd f3ans;

IDVARIABLE = f1caso;

MISSING = ALL (999.00);

CLASSES c1(4) c2(4) c3(4);

ANALYSIS:

ESTIMATOR=MLR;

TYPE = MIXTURE;

STARTS = 100 20;

MODEL: %OVERALL%

*! Measurement invariance is tested before specifying*

*! the autoregressive relationships between latent variables.*

*! c2 ON c1; ! Time 2 on Time 1 (first-order effect)*

*! c3 ON c2; ! Time 3 on Time 2 (first-order effect)*

*! c3 ON c1; !Time 3 on Time 1 (second-order effect)*

MODEL c1: *! Measurement model for children at T1*

%c1#1%

[f1adhd] (1); *!Constrain means to assume equal profile structures in all time points*

[f1odd] (2);

[f1ans] (3);

%c1#2%

[f1adhd] (4);

[f1odd] (5);

[f1ans] (6);

%c1#3%

[f1adhd] (7);

[f1odd] (8);

[f1ans] (9);

%c1#4%

[f1adhd] (10);

[f1odd] (11);

[f1ans] (12);

MODEL c2: *! Measurement model for children at T2*

%c2#1%

[f2adhd] (1);

[f2odd] (2);

[f2ans] (3);

%c2#2%

[f2adhd] (4);

[f2odd] (5);

[f2ans] (6);

%c2#3%

[f2adhd] (7);

[f2odd] (8);

[f2ans] (9);

%c2#4%

[f2adhd] (10);

[f2odd] (11);

[f2ans] (12);

MODEL c3: *! Measurement model for children at T3*

%c3#1%

[f3adhd] (1);

[f3odd] (2);

[f3ans] (3);

%c3#2%

[f3adhd] (4);

[f3odd] (5);

[f3ans] (6);

%c3#3%

[f3adhd] (7);

[f3odd] (8);

[f3ans] (9);

%c3#4%

[f3adhd] (10);

[f3odd] (11);

[f3ans] (12);

*!Unconstrain profile means in order to freely estimate profile structures across time points*

OUTPUT: SAMPSTAT STDYX TECH1 TECH7;

Mplus Syntax for LTA with two transition points, full measurement non invariance and no covariate (**Step 3**)

TITLE: LTA with full measurement non invariance and no covariate

DATA: FILE IS lta.dat;

VARIABLE: NAMES ARE

case gender ses

f1adhd f1odd f1ans

f2adhd f2odd f2ans

f3adhd f3odd f3ans;

USEVARIABLES

f1adhd f1odd f1ans

f2adhd f2odd f2ans

f3adhd f3odd f3ans;

IDVARIABLE = case;

MISSING = ALL (999.00);

CLASSES

c1(4) c2(4) c3(4);

ANALYSIS:

ESTIMATOR=MLR;

TYPE = MIXTURE;

STARTS = 100 20;

MODEL: %OVERALL%

c2 ON c1; *! Time 2 on Time 1 (first-order effect)*

c3 ON c2; *! Time 3 on Time 2 (first-order effect)*

*!c3 ON c1; ! Time 3 on Time 1 (second-order effect)*

MODEL c1: *! Measurement model for children at T1*

%c1#1%

f1adhd];

[f1odd];

[f1ans];

%c1#2%

[f1adhd];

[f1odd];

[f1ans];

%c1#3%

[f1adhd];

[f1odd];

[f1ans];

%c1#4%

[f1adhd];

[f1odd];

[f1ans];

MODEL c2: *! Measurement model for children at T2*

%c2#1%

[f2adhd];

[f2odd];

[f2ans];

%c2#2%

[f2adhd];

[f2odd];

[f2ans];

%c2#3%

[f2adhd];

[f2odd];

[f2ans];

%c2#4%

[f2adhd];

[f2odd];

[f2ans];

MODEL c3: *! Measurement model for children at T3*

%c3#1%

[f3adhd];

[f3odd];

[f3ans];

%c3#2%

[f3adhd];

[f3odd];

[f3ans];

%c3#3%

[f3adhd];

[f3odd];

[f3ans];

%c3#4%

[f3adhd];

[f3odd];

[f3ans];

OUTPUT: SAMPSTAT STDYX TECH1 TECH7 TECH15;

Mplus Syntax for LTA with two transition points, full measurement non invariance and covariates (**Step 4**)

TITLE: LTA with full measurement non invariance and covariates

DATA: FILE IS lta.dat;

VARIABLE: NAMES ARE

case gender ses

f1adhd f1odd f1ans

f2adhd f2odd f2ans

f3adhd f3odd f3ans;

USEVARIABLES

f1adhd f1odd f1ans

f2adhd f2odd f2ans

f3adhd f3odd f3ans

boy highses;

IDVARIABLE = case;

MISSING = ALL (999.00);

CLASSES c1(4) c2(4) c3(4);

DEFINE:

If (gender eq 2) THEN boy = 0;

If (gender eq 1) THEN boy = 1;

DEFINE:

If (grses eq 1) THEN highses = 0;

If (grses eq 2) THEN highses = 1;

ANALYSIS:

ESTIMATOR=MLR;

TYPE = MIXTURE;

STARTS = 100 10;

STITERATIONS = 20;

MODEL: %OVERALL%

c1 c2 c3 ON boy highses; *!Time classes regressed on covariates (gender, family SES)*

c2 ON c1; *! Time 2 on Time 1 (first-order effect)*

c3 ON c2; *! Time 3 on Time 2 (first-order effect)*

MODEL c1: *! Measurement model for children at T1*

%c1#1%

[f1adhd];

[f1odd];

[f1ans];

%c1#2%

[f1adhd];

[f1odd];

[f1ans];

%c1#3%

[f1adhd];

[f1odd];

[f1ans];

%c1#4%

[f1adhd];

[f1odd];

[f1ans];

MODEL c2: *! Measurement model for children at T2*

%c2#1%

[f2adhd];

[f2odd];

[f2ans];

%c2#2%

[f2adhd];

[f2odd];

[f2ans];

%c2#3%

[f2adhd];

[f2odd];

[f2ans];

%c2#4%

[f2adhd];

[f2odd];

[f2ans];

MODEL c3: *! Measurement model for children at T3*

%c3#1%

[f3adhd];

[f3odd];

[f3ans];

%c3#2%

[f3adhd];

[f3odd];

[f3ans];

%c3#3%

[f3adhd];

[f3odd];

[f3ans];

%c3#4%

[f3adhd];

[f3odd];

[f3ans];

OUTPUT: SAMPSTAT STDYX SVALUES TECH1 TECH7 TECH15;
